# Sustained acoustic medicine as a non-surgical and non-opioid knee osteoarthritis treatment option: a health economic cost-effectiveness analysis for symptom management

**DOI:** 10.1186/s13018-020-01987-x

**Published:** 2020-10-19

**Authors:** Thomas M. Best, Stephanie Petterson, Kevin Plancher

**Affiliations:** 1 Department of Orthopedics, UHealth Sports Medicine Institute, University of Miami, Coral Gables, FL USA; 2 Orthopaedic Foundation, Stamford, CT USA; 3 Department of Orthopaedics, Albert Einstein College of Medicine, New York, NY USA; 4 Department of Orthopaedics, Weill Cornell Medical College, New York, NY USA; 5 Plancher Orthopaedics & Sports Medicine, New York, NY USA

**Keywords:** Osteoarthritis, Sustained Acoustic Medicine, Physical therapy, Socio-economical impact, Cost-effect analysis, Health care cost

## Abstract

**Background:**

Patients diagnosed with osteoarthritis (OA) and presenting with symptoms are seeking conservative treatment options to reduce pain, improve function, and avoid surgery. Sustained acoustic medicine (SAM), a multi-hour treatment has demonstrated improved clinical outcomes for patients with knee OA. The purpose of this analysis was to compare the costs and effectiveness of multi-hour SAM treatment versus the standard of care (SOC) over a 6-month timeframe for OA symptom management.

**Methods:**

A decision tree analysis was used to compare the costs and effectiveness of SAM treatment versus SOC in patients with OA. Probabilities of success for OA treatment and effectiveness were derived from the literature using systematic reviews and meta-analyses. Costs were derived from Medicare payment rates and manufacturer prices. Functional effectiveness was measured as the effect size of a therapy and treatment pathways compared to a SOC treatment pathway. A sensitivity analysis was performed to determine which cost variables had the greatest effect on deciding which option was the least costly. An incremental cost-effectiveness plot comparing SAM treatment vs. SOC was also generated using 1000 iterations of the model. Lastly, the incremental cost-effectiveness ratio (ICER) was calculated as the (cost of SAM minus cost of SOC) divided by (functional effectiveness of SAM minus functional effectiveness of SOC).

**Results:**

Base case demonstrated that over 6 months, the cost and functional effectiveness of SAM was $8641 and 0.52 versus SOC at: $6281 and 0.39, respectively. Sensitivity analysis demonstrated that in order for SAM to be the less expensive option, the cost per 15-min session of PT would need to be greater than $88, or SAM would need to be priced at less than or equal to $2276. Incremental cost-effectiveness demonstrated that most of the time (84%) SAM treatment resulted in improved functional effectiveness but at a higher cost than SOC.

**Conclusion:**

In patients with osteoarthritis, SAM treatment demonstrated improved pain and functional gains compared to SOC but at an increased cost. Based on the SAM treatment ICER score being ≤ $50,000, it appears that SAM is a cost-effective treatment for knee OA.

## Introduction

Osteoarthritis (OA) is the most common type of arthritis affecting over 63 million adults in the USA annually [1]. The most common presentation of OA is in the knee, occurring in 10–13% of men and women over the age of 60 with an estimated 14 million people having this condition [2, 3]. The prevalence of OA is projected to increase as people become more obese and the population skews towards older age. OA is characterized by joint inflammation with chronic pain and decreased function of the joint as stiffness and swelling limit mobility.^4^ In turn, quality of life and productivity are adversely affected [3, 4].

Pain from OA is one of the key reasons why patients seek medical care and generally precedes disability/loss of function [5]. As the pain worsens, the treatments first focus on pain relief and the inflammatory aspects of the disease [6]. In recent years, there has been an increasing emphasis on non-pharmacologic therapies to treat the symptoms of OA including physical therapy, exercise, massage, and ultrasound which for most are not as easily accessed due to patients lacking the resources or ability to do so, ultimately contributing to worse pain and greater disability [7]. Recent recommendations by the American College Rheumatology (ACR)/Arthritis Foundation for treating knee OA call for a comprehensive plan including the above therapies which may be used in sequence, again depending upon pain severity [8].

The effectiveness of ultrasound as a therapy for OA-related pain and disability has been substantiated in a number of systematic reviews and meta-analyses [9–12]. However, one of the challenges with current ultrasound delivery is that reimbursement requires administration via constant attendance by a care provider in a professional setting, generally for a short duration of time (5–10 min) over approximately a 30-day period. This is likely due to the types of high quality studies that have examined this condition and associated treatment protocols as well as payer policies, which only cover for direct one-on-one constant attendance by a provider [13–15]. The policies and in-office treatment requirement prohibits daily treatment in the clinical setting for many patients and medical professionals. Despite the short duration of in-clinic ultrasound treatment, residual positive effects on pain and function with this modality appear to last upwards of 1 year [16, 17]. Recently, daily, multi-hour sustained acoustic medicine (SAM), low-intensity, long-duration wearable ultrasound has been found to provide effective pain relief and functional improvement in patients with knee OA [18–23].

It is with the above in mind that a cost-effectiveness analysis was undertaken to examine accepted treatment pathways in using SAM ultrasound versus standard of care (SOC) in the relief of knee OA pain and improvement of function over a 6-month period. The reason 6 months was chosen is that all generally accepted therapies for pain relief could be evaluated, with the intention that therapies that improve function could then be evaluated. The purpose was to examine the overall direct costs for care in using one therapy versus another and to examine the outcome of the patient. Additionally, the SAM ultrasound therapy system that can be used in the home over long durations without direct one-on-one provider contact was evaluated. To our knowledge, such an analysis has not been previously conducted.

## Methods

The patients evaluated in the model presented with pain characterized as intense, unpredictable, and emotionally draining resulting in the avoidance of physical activity [24]. In other words, the pain was severe enough such that there was an avoidance of physical therapy, which has consistently been shown to improve function [25]. This type of pain is also distressing enough that it affects the person’s quality of life, limiting activities of daily living and other recreational activities [16]. Additionally, these patients were < 65 years of age (i.e., non-Medicare).

The clinical guidelines used for treatment of knee OA in this assessment were the 2019 ACR/Arthritis Foundation guidelines for the management of OA of the hand, hip, and knee [8]. All therapies recommended by the ACR were used in the model. However, one therapy that was not recommended by the ACR was also used, hyaluronic acid (HA) injections (ACR has a conditionally recommended against for HA) [8]. Inclusion of HA use was due to the identification of a 2009 Cochrane Review on viscosupplementation where HA was supportive of its use for relieving pain [25].

**Fig. 1:**
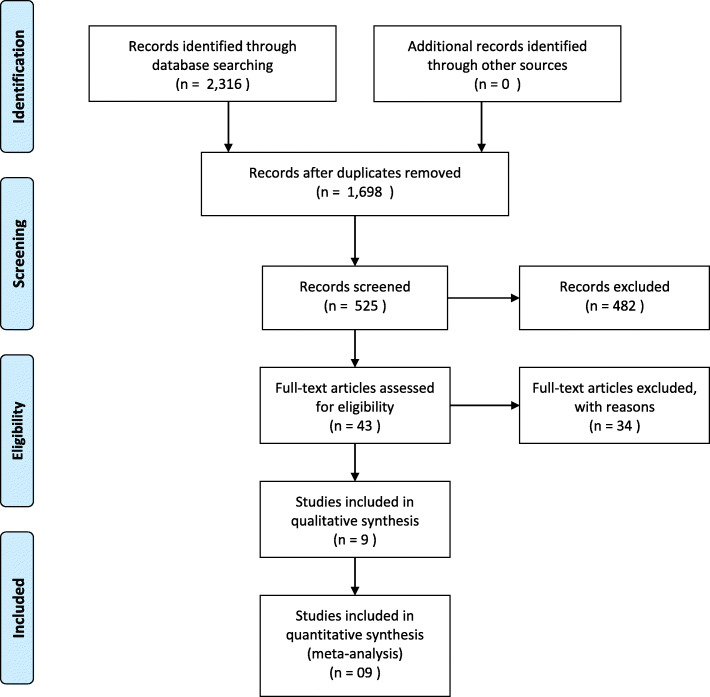
PRISMA flow diagram for identification, screening, eligibility, and included articles in health economic modeling data

Identification of the highest quality of evidence was used in the evaluation of outcomes for pain relief and function for all therapies. The types of studies identified therefore were systematic reviews and meta-analyses. PubMed and Cochrane Library were the databases searched with the key words used for identifying studies being:

((((((((((((((systematic) AND review) AND meta-analysis) AND pain) AND knee) AND osteoarthritis) AND outcome)) AND function)) AND random*) AND control*) AND trial)) AND NSAID–hits 292

((((((((((((((((systematic) AND review) AND meta-analysis) AND pain) AND knee) AND osteoarthritis) AND outcome)) AND function)) AND random*) AND control*) AND trial)))) AND physical therapy–hits 1,174.

((((((((((((((((((systematic) AND review) AND meta-analysis) AND pain) AND knee) AND osteoarthritis) AND outcome)) AND function)) AND random*) AND control*) AND trial)))))) AND hyaluronic acid–hits 186

(((((((((((((((((((((((systematic) AND review) AND meta-analysis) AND pain) AND knee) AND osteoarthritis) AND outcome)) AND function)) AND random*) AND control*) AND trial)))))))))) AND steroid) AND injection*–hits 211

((((((((((((((((((((systematic) AND review) AND meta-analysis) AND pain) AND knee) AND osteoarthritis) AND outcome)) AND function)) AND random*) AND control*) AND trial)))))))) AND ultrasound – hits 453. Figure 1 identifies the process of identifying the systematic review and meta-analyses used in the current analysis.

In order to simplify the model and not introduce confounding therapies, the assumption was made that therapies were used sequentially for both SAM treatment and SOC based on ACR care guidelines. In other words, if a therapy improved upon pain enough (with pain being the limiting factor for engaging in PT) such that PT could be initiated, it ended the decision to use another pain therapy. However, if pain was not improved, another modality was employed. The probability of improvement in pain such that PT could be initiated was identified in the systematic reviews and meta-analyses identified in the searches [25–28].

Costs were derived from manufacturer pricing [29], reimbursement rates over last 3 years for the SAM device including federal agencies TRICARE Military and VA Health System [30], and from 2020 National Medicare rates adjusted by the ratio of Medicare payments to commercial payments in order to obtain the commercial rates [31, 32].

Lastly, the statistically significant effect sizes for a therapy in improving pain relief and function were identified in the systematic reviews and meta-analyses [27, 33, 34]. The effect size is a simple way of quantifying the size of a difference between 2 groups. An effect size of greater than 0.5 generally means that the difference is important and can be observable which was used in the model [35]. It was further assumed that when PT was initiated (based on pain improvement), it was continued for the duration of the 6-month analysis as PT has been shown to have a positive effect on function over time [36]. The number of sessions and duration (e.g. dosing) was derived from the medical literature whereby the largest effect could be identified [36].

Pain and function were established endpoints of the model as they are arguably of the most important outcomes in patients with knee OA. The American Academy of Orthopedic Surgeons (AAOS) has stated that: “the quality and success of interventions to treat OA should be assessed based on outcomes deemed to be of importance to the patient. ”[37]. The measure of pain and function in knee OA has also been established as part of the physician quality reporting system (PQRS #109) [38].

The incremental cost-effectiveness ratio (ICER) was used in measure improvement of pain and function, and has been used in the past in knee OA for determining the ICER of a therapy [39]. Pain and function have been recognized as important outcome measures by the Institute of Clinical Economic Review, a leading organization in the area of value analysis of therapies [40]. For this particular analysis, the ICER was calculated as follows: (cost of SAM treatment minus cost of SOC)/(functional effectiveness of SAM minus functional effectiveness of SOC).

**Fig. 2:**
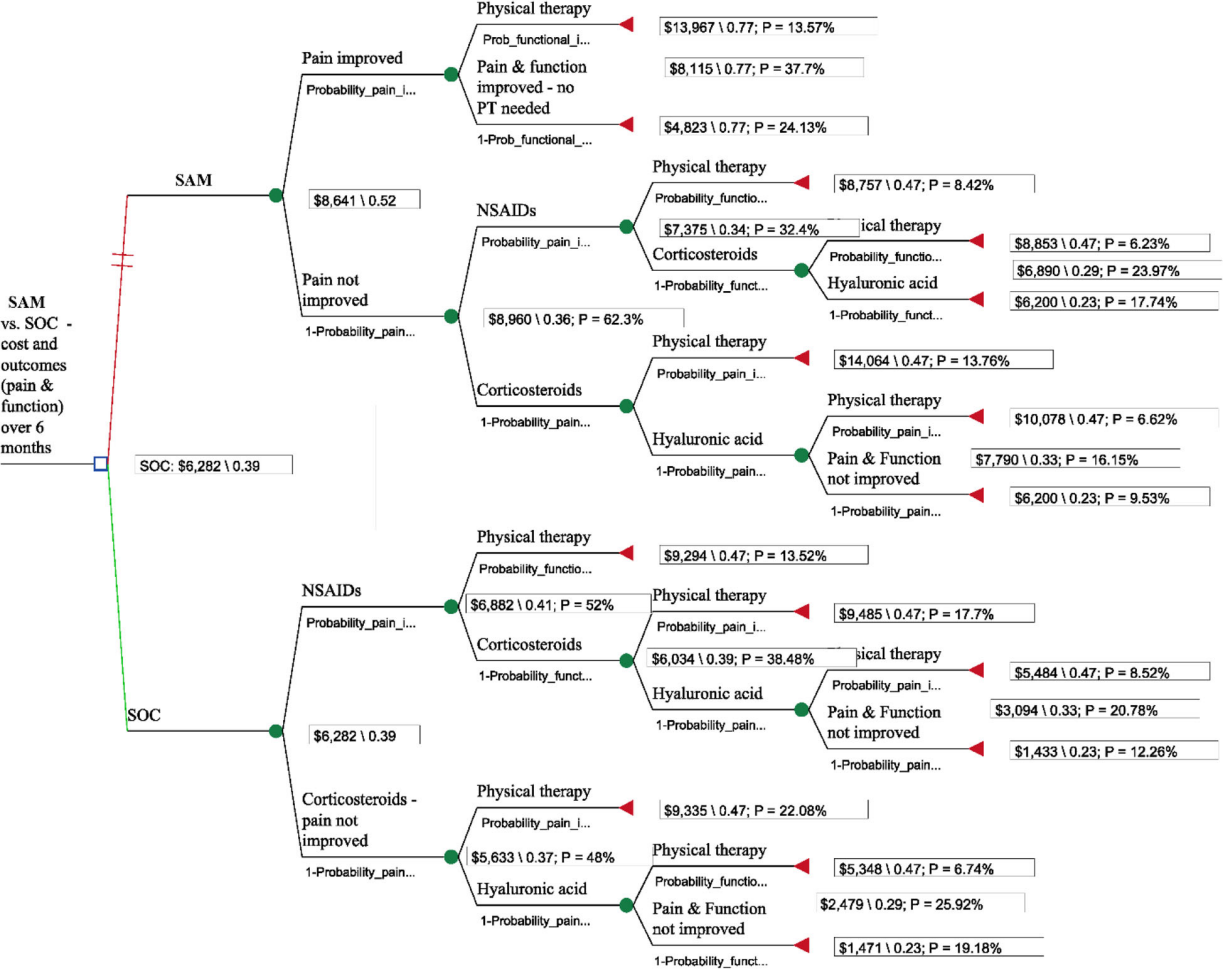
Decision tree model of cost, effect size, and probability for the 6-month management of knee osteoarthritis. Physical therapy combined with SAM provides superior non-invasive and drug-free treatment versus the SOC care pathway for patients

Tree Age Pro Healthcare 2020 software (TreeAge Software Inc., Williamstown, MA, USA) was used in the analysis. The variables, distributions and equations used in the model are found in Appendix 1 and 2. The decision tree is found in Fig. 2. Additionally, a tornado plot was employed to determine which variables in the model had the greatest effect on costs such that one therapy was found to be less expensive than the other. These variables were then examined individually in sensitivity analysis to identify the value at which one therapy became more expensive relative to the other and whether this value was realistic in everyday practice. An ICER scatterplot was also evaluated whereby the variables in the model were randomly varied 1000 times using the low and high values as identified in the variables/distributions (Appendix 1).

Lastly included in the model, and as mentioned above, a wearable, long duration, continuous home-use ultrasound system that is self-administered, and capable of delivering 18,720 J of ultrasound therapy for up to 4 h/day generating vigorous deep musculoskeletal diathermy Δ8 °C at 1 cm, Δ6 °C at 3 cm, Δ3 °C at 5 cm; and allows a patient to utilize ultrasound therapy outside of a medical facility (Sustained Acoustic Medicine [SAM] device, FDA Approval # K191568, ZetrOZ Systems, LLC, Trumbull, CT, USA). This device has been used safely and effectively in the treatment of knee OA [20–23] chronic myofascial pain [41–43], tendinopathies [44–47], and for healing various musculoskeletal injuries [19, 22, 44–47]. Markov modeling (1000 iterations) was used to examine on a percentage basis were SAM treatment would be a more effective and more costly therapy versus SOC.

## Results

In the initial knee OA care pathway of the model shown in Fig. 2, the cost and effect size on pain and function over 6 months was $8641 and 0.52, respectively for SAM treatment and $6282 and 0.39, respectively for SOC. Thus, the ICER for the use of SAM was $18,146 ($8641 less $6282)/(0.53 less 0.39). In the later stage, knee OA care pathway with response to SAM with the addition of PT the cost and effect size was $13,967 and 0.77, respectively, versus SOC combined with NSAIDs and PT at $9294 and 0.47, respectively. The later care pathway SAM ICER value was $15,576 ($13,967 less $9,294)/(0.77 less 0.47).

**Fig. 3:**
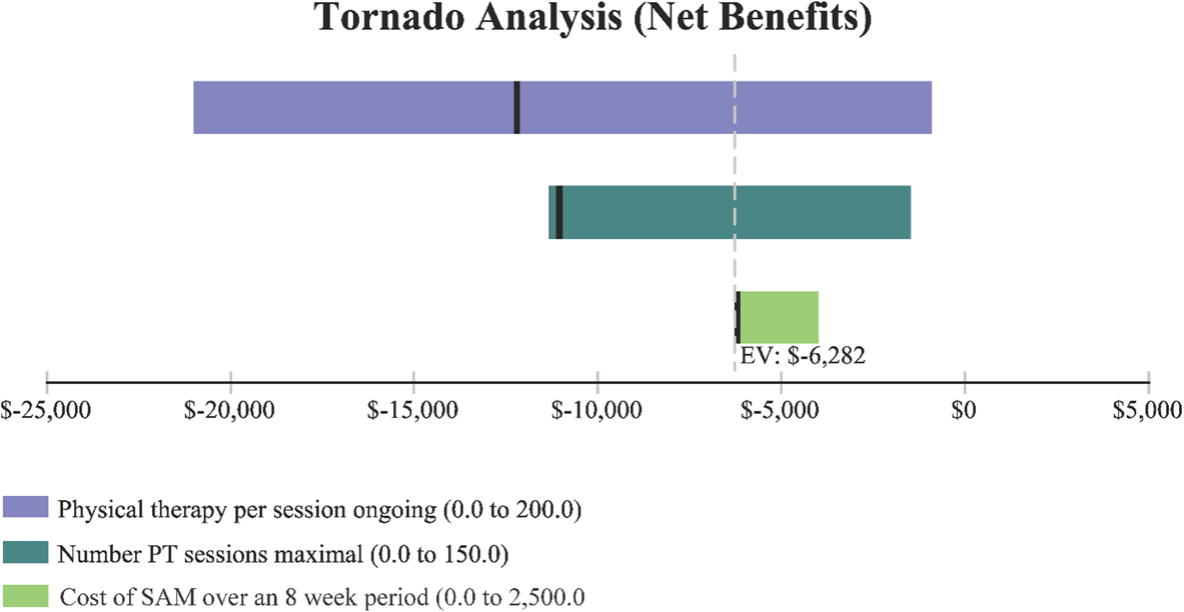
Model variables with greatest effect on cost of care pathway and the ranging parameters

A tornado plot (Fig. 3) identified the following variables which had the greatest effect on cost (i.e., in identifying SAM as the least costly option) including the cost of SAM, cost of a 15-min PT session, and the number of PT sessions. Additional sensitivity analysis identified the following variables and their threshold values were SAM treatment becomes the less costly alternative (Table 1).

**Table 1:** Cost-effectiveness model variables where SAM treatment of knee osteoarthritis becomes less costly. Each model variable is independent based on the base model assumptions

Variable	Value at which SAM treatment becomes the less costly alternative	Figure
SAM treatment cost	< $2276	Fig. 4
Cost for PT per session	> $88	Fig. 5
Number of 15 min PT sessions	> 144	Fig. 6

**Fig. 4:**
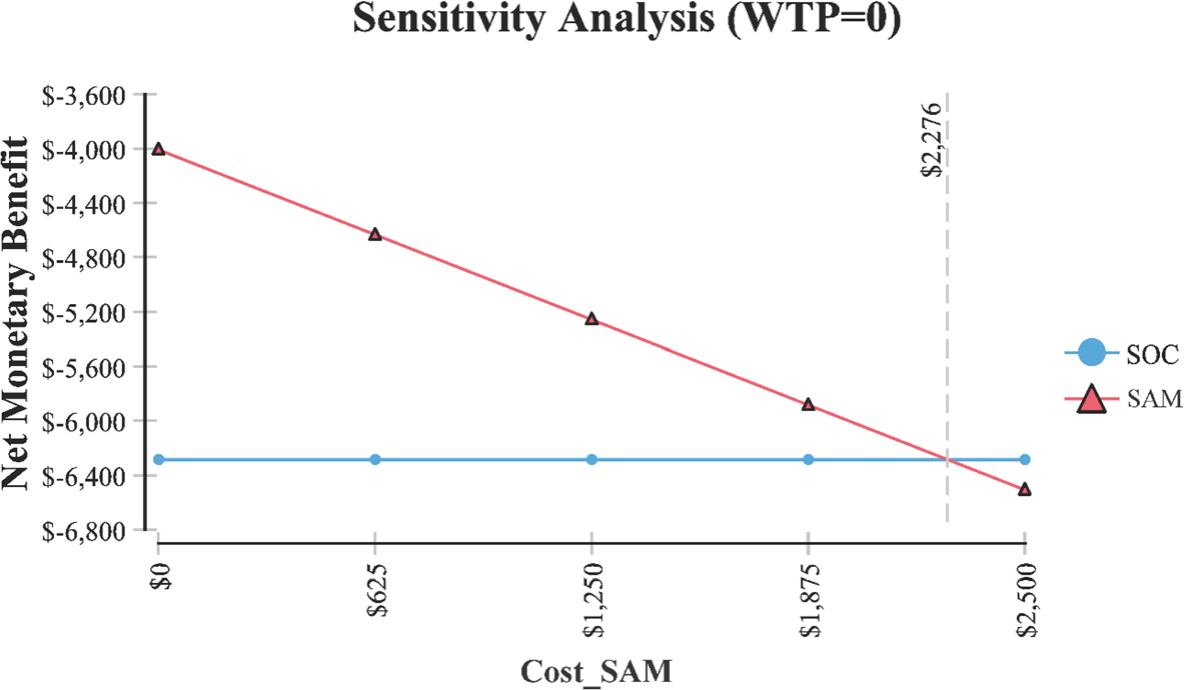
Sensitivity analysis on SAM cost where treatment is less expensive option to SOC

**Fig. 5:**
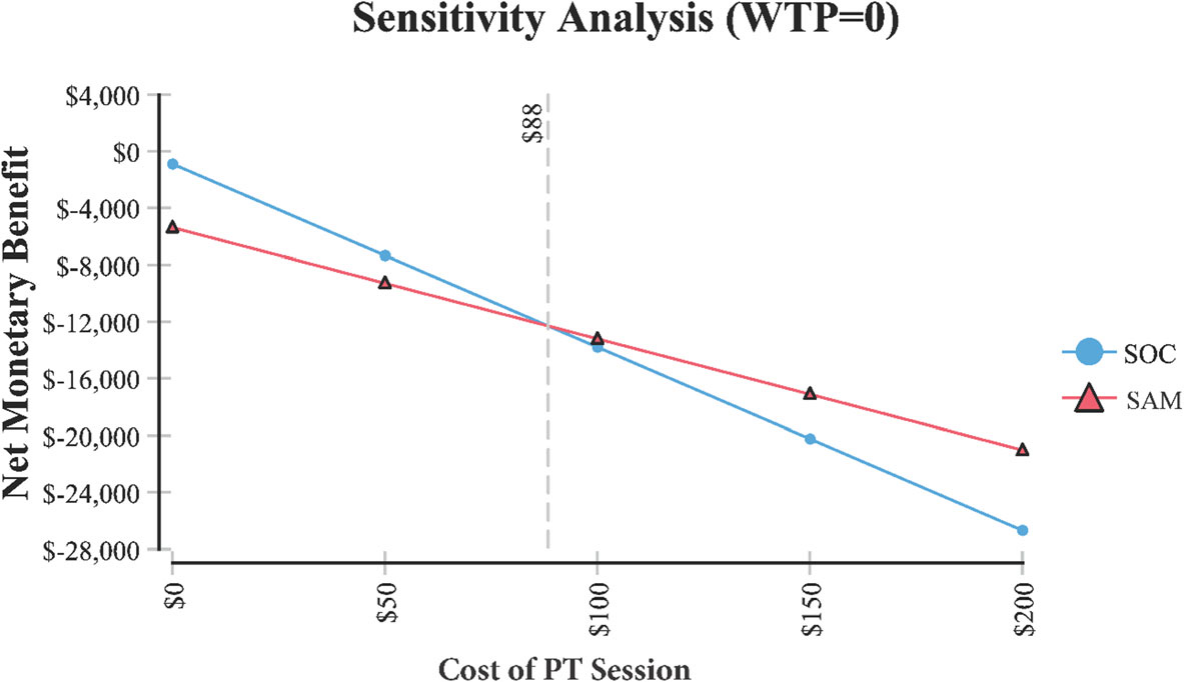
Sensitivity analysis relative to PT session costs, where SAM treatment costs less than SOC

**Fig. 6:**
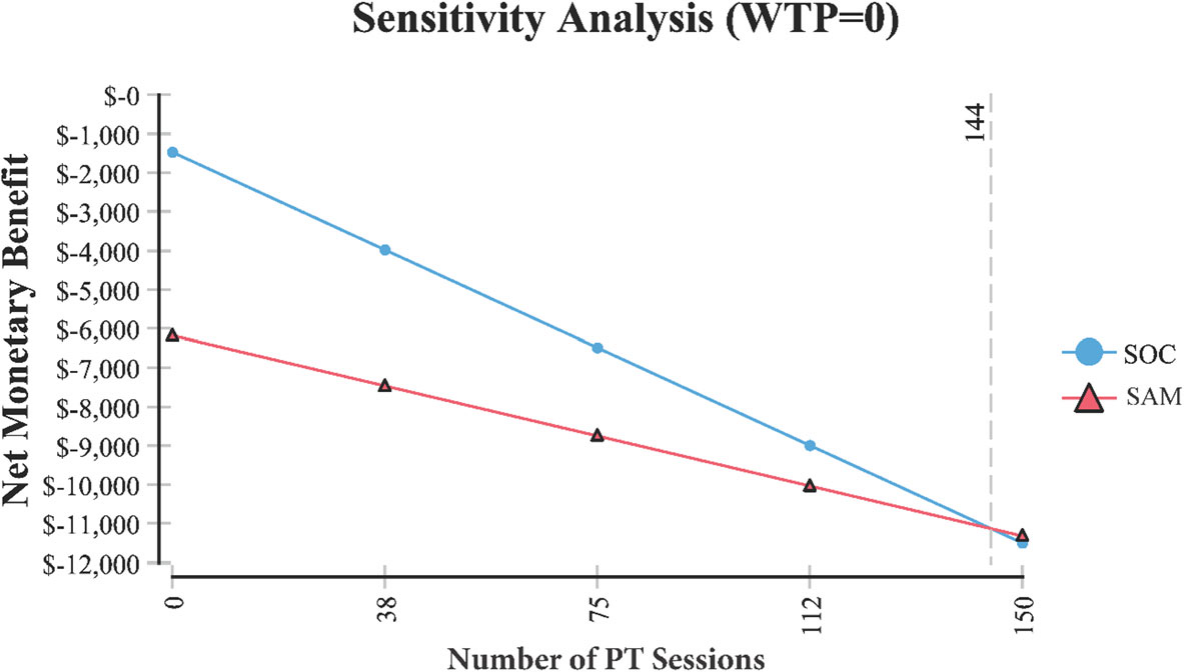
Sensitivity analysis relative to the number of PT sessions where SAM treatment costs less than SOC

**Fig. 7:**
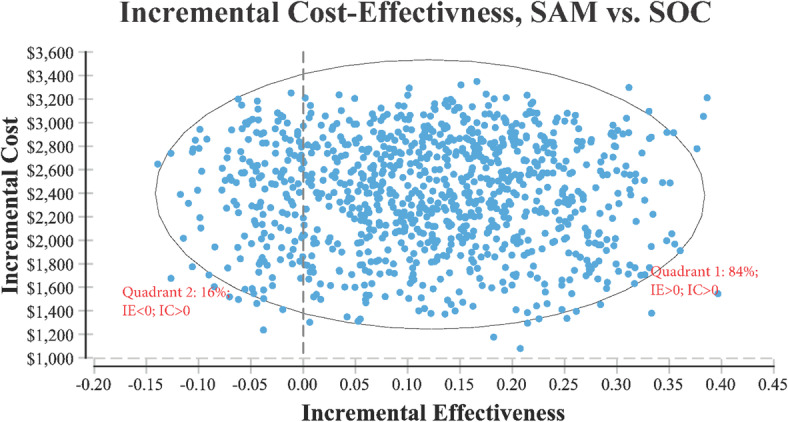
Markov modeling scatter plot of cost effectiveness for SAM treatment versus SOC

In order for SAM treatment to be the less expensive option relative to SOC, it would need to cost < $2276 (Fig. 4). The price of SAM treatment used in the model is $4635 (average reimbursement price of the unit). For SAM treatment to be the less expensive option, the cost of PT would need to be > $88 per 15-min session (Fig. 5). It is assumed in the model that the cost per 15-min PT session was $41.80 and that a patient would receive anywhere from 2 to 4, 15-min sessions per visit. Or in order for SAM treatment to be the less expensive option, > 144 total sessions (15-min per session) would need to occur (Fig. 6). Lastly, Fig. 7 demonstrates in Markov modeling that 84% of the time SAM treatment was more effective in pain and function outcomes for knee OA versus SOC pathway, and a more costly therapy versus SOC over 6 months of treatment. The centroided of the Markov model demonstrates an incremental cost of + $2400 and effect of + 0.12 for SAM treatment of knee OA.

## Discussion

The major finding from our analysis was that the home-use SAM device for the treatment of OA pain and subsequent improvement in function and quality of life was cost-effective with an ICER between $15,576 and $18,146. The calculated SAM ICER value range falls within accepted cost-effectiveness threshold values in determining whether a therapy is cost-effective. The Institute for Clinical and Economic Review considers ICERs of $50,000–$100,000 high value care [40]. Payer organizations such Premera Blue Cross of Washington identify therapies as cost-effective if they are between the values of $10,000–$50,000 [48]. Specialty societies such as the American College Cardiology identify high value care as an ICER < $50,000 [48]. Lastly, cost-effectiveness systematic reviews for the surgical management of OA including procedures covered by Medicare have ICER values $10,000–$100,000 [49].

Reduction of pain and increased function are important outcomes for patients with knee OA and are commonly used as endpoints in studies examining therapies for treating this disease [49]. These endpoints are observable, measurable, and most relevant to the patient [37]. It has also been identified that function is highly correlated with quality of life (QoL) in patients with knee OA [50–52], another endpoint evaluated in our ICER analyses. Thus, the use of function as well as pain as an outcome in ICER appears to be a good proxy for QoL and are commonly used [53].

Widespread use of traditional ultrasound in treating both pain and improving function of patients is hampered by the requirement that it has traditionally been limited to us in a clinical setting, is of short duration, and must be applied by a clinician with direct provider to patient contact [13–15] These limitations are further enhanced by the recent 2020 pandemic which has reduced patient access to clinical practices and has resulted in new remote care policies being issued by insurance carriers and governmental agencies [54, 55]. Other limitations include specialty societies and payers in making recommendations for ultrasound treatment of knee OA or lack thereof [8, 56–58]. Unfortunately, some payer coverage and utilization policies have not been updated for over 10 years, failing to account for recent, high quality evidence in establishing coverage determinations [13, 14, 58]. Additionally, there appears to be an issue with the use of office-based ultrasound devices causing harm (e.g., burning and overexposure) if the ultrasound head is not moved constantly over the affected site [18, 19]. However, recent high quality studies of FDA-cleared home use of low intensity long duration SAM ultrasound for symptomatic relief of knee OA have demonstrated similar efficacy to clinical setting use [20]. Two earlier pilot studies and two RCTs demonstrated similar findings for this type of SAM treatment for knee OA [21, 22]. Long duration continuous SAM ultrasound has demonstrated good safety with unattended use in the home and work setting [18, 20–23, 41–44, 46, 59–61].

Recent studies have identified that greater than 25% of patients with knee OA use opioids and that increased pain severity increases their use [62, 63]. Further, it has been found that the use of opioids for pain relief significantly increases the downstream costs (either alone or concomitantly with PT) vs. PT only [64]. Lastly, what is interesting to note is that the timing of non-pharmacological therapies (i.e., early vs. later in the diagnosis of knee OA) results in a lower usage of opioids, likely due to a patient experiencing pain reduction [65]. A non-invasive therapy such as SAM used early in the diagnosis and in the home could lessen the use of opioids, and possibly help with the current epidemic of overuse/over prescribing [66]. Since opioid treatment was not considered as an option in the current analysis (only topical NSAIDs), it is a limitation of this analysis. For this very reason, however (i.e., minimizing the need for opioid use), SAM treatment should be considered a viable, prescribed, and covered home therapy.

It is interesting to note from the sensitivity analysis that for SAM treatment to be a less costly alternative compared to SOC, it would need to be less than $2276. The model uses a price of $4635, which is the reimbursement price of the equipment evaluated in the model. A patient owning the equipment could lead to more extended use in the home, alleviating the need to visit a clinician and the costs associated with travel to and from a clinical setting for the patient and; additional follow-on care longer term.

The assumed cost for PT used in the model was $41.80 per 15 min. It was found in our sensitivity analysis that for SAM to be the less expensive option that this cost per 15 min would need to be greater than $88. Therefore, SAM may be the less expensive option for some private payers that pay at the $88 or more rate.

Finally, the model found that SAM combined with PT provided superior outcome for the patient without the use of drugs or injections, and the modest incremental cost of care could provide significant cost savings for a life-long progressing disease; the ICER for SAM + PT versus NSAIDs +PT was found to be $15,576.

### Limitations

Medicare rates were assumed to be 75% of the commercial private payer rate—i.e., for every $1 spent with Medicare, the commercial rate would be $1.33. Commercial rates may be higher than this in some parts of the country based on negotiated rates between providers and payers, upwards of 100% higher with certain payers [67].

Costs used in the analysis were based off accepted treatment guidelines as identified by the American College of Rheumatology and were not prospectively captured [8]. As well, costs were assumed to be national averages and thus would vary by region of the country.

As mentioned above, opioid use was not evaluated, only topical NSAIDs. Topical NSAIDs were shown to be effective in relieving pain in identified systematic reviews and meta-analysis, and were used based on their short term positive effects. Longer term, some of these patients may be prescribed opioids. The risks of addiction to opioids (and associated costs and outcomes) were not evaluated. This could be evaluated in future studies.

No complication rates or costs of them were assumed for therapies used in the analysis. Considering many were very low, the cost and effect were assumed to be de minimize.

## Conclusion

Based on accepted thresholds in determining the cost-effectiveness of therapies in treating knee OA, SAM, wearable home-use continuous long duration ultrasound, appears to be a cost-effective therapy and should be considered when treating patients with knee OA.

## Supplementary information

**Additional file 1:.** Variable distributions

**Additional file 2:.** Equations used in model

## Data Availability

https://pubmed.ncbi.nlm.nih.gov/ (pubmed.gov) https://www.cochranelibrary.com/ (Google Scholar) https://scholar.google.com/ (Cochrane Library)

## Abbreviations

OA: Osteoarthritis
SAM: Sustained Acoustic Medicine
SOC: Standard of care
ICER: Incremental cost effectiveness ratio
PT: Physical therapy
ACR: America College Rheumatology
NSAID: Non-steroidal anti-inflammatory drug
VA: Veteran affairs
FDA: Federal Drug Administration
QoL: Quality of life
